# Advances in Etiology and Prevention of Capsular Contracture After Breast Implantation

**DOI:** 10.1007/s00266-024-04500-5

**Published:** 2024-11-25

**Authors:** Dan-Dan Li, Nan Lan, Ping Zhao, Yi-Yin Tang

**Affiliations:** 1 The Second Department of Breast Surgery, The Third Affiliated Hospital of Kunming Medical University, No. 519 of Kunzhou Street, Xishan District, Kunming, 650000 China; 2 The First Department of Breast Surgery, The Third Affiliated Hospital of Kunming Medical University, Xishan District, No. 519 of Kunzhou Street, Kunming, 650000 China

**Keywords:** Breast implants, Implant capsular contracture, Protective agents, Disease prevention, Secondary prevention

## Abstract

Capsular contracture (CC) is one of the most common complications of breast implant usage in breast augmentation or reconstruction. The CC approach can cause breast hardening, pain, and varying degrees of deformity, affecting the quality of life of patients. Considerably, it has become one of the most common reasons for frequent surgeries. Nonetheless, the etiology and pathogenesis of CC remain unclear. Moreover, there exist still a lot of uncertainties regarding prevention and treatment measures. In this article, we present discussions on the research status of the etiology, pathogenesis, prevention, and treatment measures of CC. In summary, this study provides a reference for further research on CC and clinical use.

*Level of Evidence V* This journal requires that authors assign a level of evidence to each article. For a full description of these Evidence-Based Medicine ratings, please refer to the Table of Contents or the online Instructions to Authors www.springer.com/00266.

## Introduction

Capsular contracture (CC) has remained one of the most prevalent complications following breast implant surgeries, including for aesthetic purposes or postmastectomy reconstruction [1, 2]. Typically, the formation of a fibrotic capsule around the implant often results in hardening, pain, and breast deformity, requiring additional surgical intervention [3]. Despite its frequent occurrence, the underlying causes of CC condition are not yet fully explored, making it highly challenging for both clinicians and patients. Several factors have been identified as potential contributors, such as immune responses to the implant, bacterial contamination, implant surface characteristics, and patient-specific factors like genetic predisposition [4].

Several advancements in breast implant technology have contributed to a reduction in CC occurrence rates, such as the development of textured implants and acellular dermal matrices (ADM) [5]. Nevertheless, the challenges remain to be comprehensively eradicated. Several advancements have been evidenced in the continued exploration of therapeutic strategies [6–9]. These innovative therapeutic strategies range from improved surgical techniques to surface modifications of implants, aiming to enhance biocompatibility and reduce fibrotic responses.

This review aims to provide an in-depth overview of the recent progress in understanding the etiology and prevention of CC. The data were obtained from the Web of Science Core Collection, covering literature from January 1, 2013, to September 22, 2023, with search terms focusing on CC in breast reconstruction, risk factors, macrophages, and preventive measures. The existing studies are analyzed to highlight both the known mechanisms involved in CC and the emerging strategies for minimizing its occurrence, thus offering a roadmap for future research and clinical practice in this field.

## Research Methods and Data Sources

The CiteSpace software and the log-likelihood ratio (LLR) algorithm were used for literature analysis. Initially, the datasets were obtained from the Web of Science (WoS) Core Collection. Accordingly, the literature search was performed using the following strategy: SU = [(capsular contracture *) AND (breast reconstruction *)] OR SU = [(capsular contracture *) AND (risk factors *)] OR SU = [(capsular contracture *) AND (macrophages *)] OR SU = [(capsular contracture *) AND (preventive measures *)], ranging from January 1, 2013, to September 22, 2023. Further, the research hotspots were visually reflected by the visual analysis of the intercountry cooperation network, keyword co-occurrence clustering analysis, keyword burst, and timeline attributes. Finally, the evolutionary and frontier trends of the clustered analyses were explored.

## Results

**Fig. 1:**
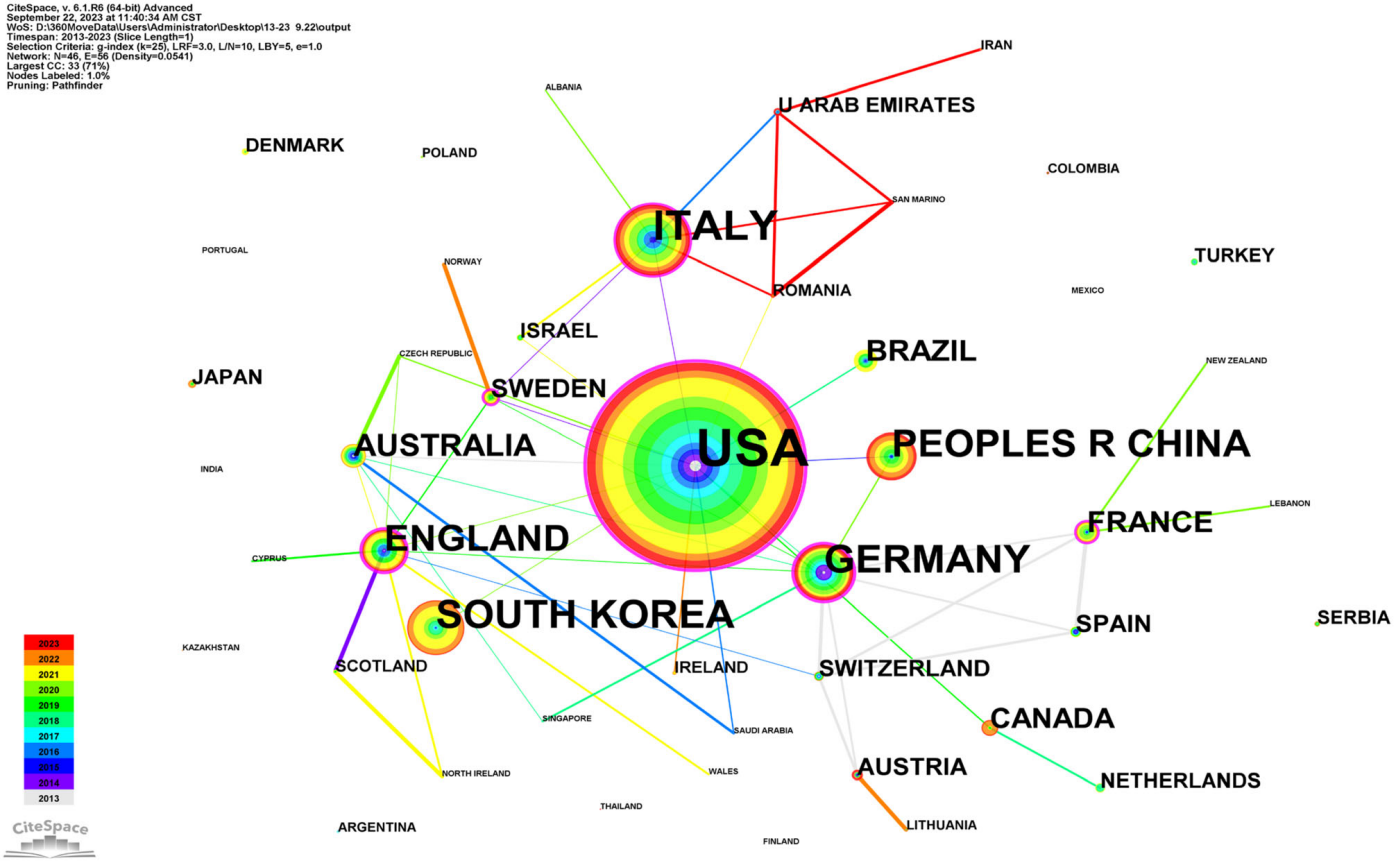
The image shows the knowledge map of countries' cooperation networks

**Fig. 2:**
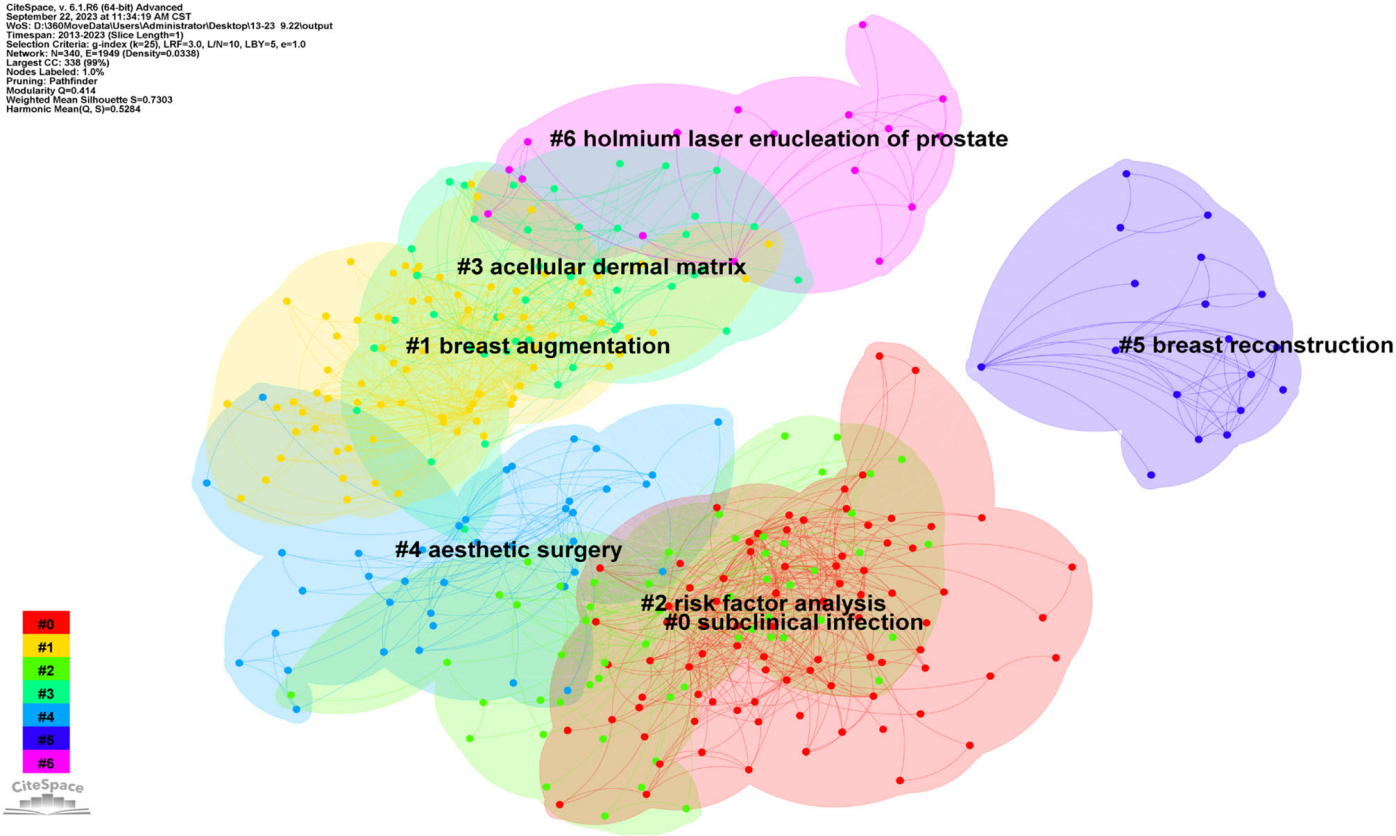
The image shows the keyword cluster map

**Fig. 3:**
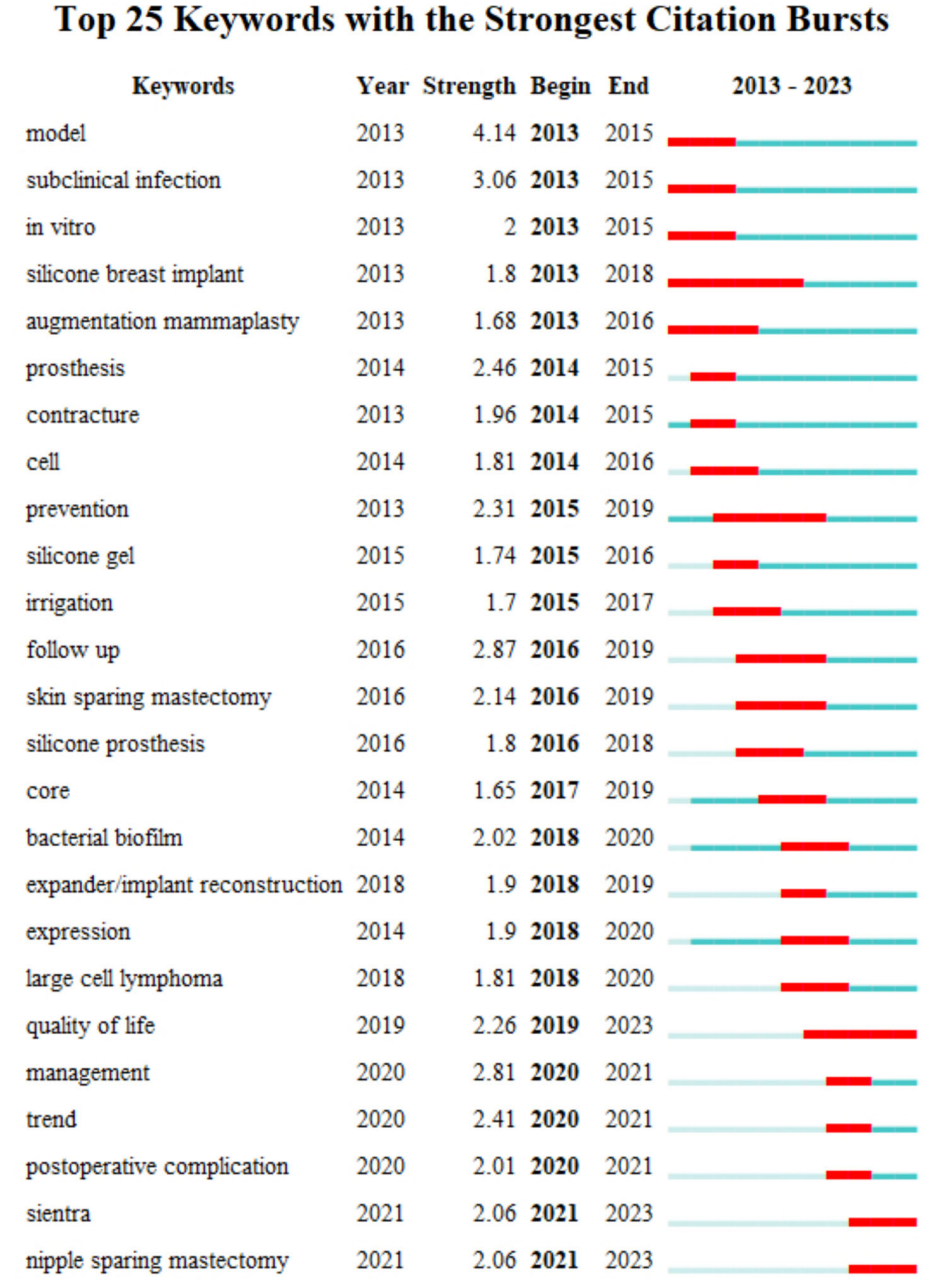
The image shows the keyword burst details

Notably, the distribution of the various countries was analyzed using a country as the node type. The visualization map of the intercountry cooperation network was obtained with 46 nodes (*N*) and 56 connecting lines (*E*) and an overall network density of 0.0541 (Fig. 1). The results indicated that the USA was the most studied country, followed by Italy and Germany. The number of publications was far larger in the USA compared to other countries. From the perspective of centrality, a positive correlation was observed between the number of publications and centrality in most countries, indicating relatively mature research globally. In addition to countries, the research hotspots could attain the focus of attention of scholars in specific academic fields, reflecting the main issues discussed in the field over a certain period. Moreover, keywords, an important part of academic papers, can be used to study the research focus of a certain field while condensing the essence of papers. Thus, the keyword co-occurrence clustering analysis was accordingly performed to reflect the research hotspot visually. Figure 2 shows the keyword cluster map with *N* = 340, *E* = 1949, and Density = 0.033. Moreover, the values of *Q* = 0.414 and *S* = 0.7303 could indicate excellent clustering of the network structure, higher homogeneity, and better classification of different clusters, respectively. The seven clusters were dominated by "subclinical infection," "breast augmentation," and "risk factor analysis" (Fig. 2). Among these clusters, the top five clusters were mainly concentrated between 2016 and 2018, suggesting that relevant studies were mature during this period. Figure 3 displays the burst terms in the research field in the past decade, indicating that "silicone breast implant" and "prevention" were research hotspots for a long time due to the high burst rate.

The timeline mapping presented the keyword clustering of the literature on a two-dimensional timeline. Thus, researchers could substantially explore the evolutionary and frontier trends of topic clusters more intuitively, providing references for the relation between hot topics. Among them, several keywords, including "subclinical infection," "breast implant," and "surface," were the crossed keywords except those related to CC. The results suggested that CC might be related to inflammation, type of prosthesis, and implant surface morphology. Therefore, the research of the etiology, pathogenesis, prevention, and treatment measures of CC was further reviewed comprehensively to provide a reference for further research on CC and benefit clinical utilities.

## Discussion

Capsular contracture (CC) has emerged as one of the most multifactorial complications commonly observed in breast implant patients, involving a combination of immune responses, infection risks, surgical techniques, and implant characteristics. In this section, we provide an analysis of the key risk factors contributing to CC, followed by a discussion of the strategies and measures to reduce CC risk.

### Risk Factors for CC

#### Implant Surface Characteristics

Recently, several reports suggested that the textured implants showed a lower incidence of capsular contracture (CC) compared to smooth implants. In this context, the nanostructured implants showed the minimum capsular thickness, reduced collagen density, and myofibroblast infiltration, offering exceptional biocompatibility. Typically, the macrotextured implants may cause the bicapsular formation and are linked to anaplastic large cell lymphoma (ALCL). Unlikely, the nanostructured implants balance roughness and mobility, reducing the risk of CC occurrence. Thus, nanostructured implants are often considered a safer alternative to both smooth and macrotextured implants in breast augmentation [1–18]. To this end, the macrotextured implants are contaminated with a larger area of bacteria, thus promoting inflammation and the development of a rare malignancy, i.e., ALCL [19]. Several clinical reports consistently showed that implant surface texture could play a crucial role in the risk of CC occurrence. Accordingly, the smooth implants could form denser, more regular collagen fibers, increasing capsular stress, while the macrotextured implants could create more irregular fibers. Thus, the arrangement could impact capsular contracture risk, with smoother implants triggering stronger myofibroblast responses than macrotextured implants [19–22].

In a study, patients (*n* = 30) were divided into five treatment groups with different implant textures (Smooth, Poly, L-Micro, H-Micro, and Macro), in which Poly and L-Micro implant groups showed an increased tissue remodeling, reduced myofibroblast activation, and greater neovascularization, contributing to lesser fibrous and unstable capsules and significantly reducing the risk of CC over other groups. Patients with smooth implants exhibited the highest incidence of CC occurrence among the treatment groups, likely due to the formation of dense collagen around the implant [22]. In contrast to the clinical findings, textured implants in animal studies showed thicker capsules, lower collagen density, and significant differences in the expression levels of collagen type I and CD68 compared to smooth implants. The resultant discrepancy might be due to differing responses between animals and humans [23–27]. Notably, the surface morphology of the implant often affects the immune response and foreign body reaction (FBR), with microimplants inhibiting fibrosis and increasing immunosuppressive FOXP3+ T cells. In one instance, the navitoclax treatment resulted in reduced IL-17 expression and fibrosis by targeting senescent cells. Macrophages, specifically the M2 phenotype, could promote tissue repair and reduce fibrosis, in which the coarse-textured implants favoring the M2 phenotype could minimize the fibrotic response to implants. These findings highlighted the role of implant surface in modulating immune responses and fibrosis [23–27]. Further, the animal studies demonstrated that micro-smooth silicone implants showed lower levels of MMP3 and TNNT3 and higher NRG-1 compared to textured implants, with similar results in human CC. Nonetheless, there exists some argument, as some studies suggest smooth implants could reduce CC risk. In addition, the implant surface and shell hardness could impact CC incidence, with softer implants reducing collagen deposition and myofibroblast activation. Several techniques, like 3D imprinting, could create surfaces with high contact points and low roughness, promoting natural tissue interactions and potentially lowering CC risk. Despite the progress, large-scale and long-term studies are required [28–30].

#### Surgical Operation Affects CC

In addition to implant surface characteristics, surgical factors significantly affect the risk of capsular contracture (CC) occurrence, such as contamination during surgery, postoperative infections, hematomas, and foreign body residues. In a study, patients (*n* = 322) showed the highest CC incidence at periareolar incisions (5.36%), followed by vertical (3.48%) and inframammary fold incisions (1.64%), which could be due to the higher number of mammary ducts near the nipple. In another instance, several patients (*n* = 141) confirmed lower CC rates with inframammary fold incisions over periareolar incisions. In addition, subglandular implants possess a higher CC risk compared to submuscular implants [31–38]. A meta-analysis demonstrated that the submuscular IBBR could significantly reduce capsular contracture (CC) risk and prosthesis failure compared to subglandular IBBR. Contrarily, another study showed lower CC and infection rates in subglandular IBBR, especially in irradiated patients due to muscle fibrosis [39–41].

#### Infection Affects CC

Tamboto and colleagues demonstrated a strong association between microorganisms and CC, indicating the correlation between subclinical infection and biofilm formation with CC in a porcine model. The bacterial contamination could lead to biofilm formation on implant surfaces, increasing the risk of CC occurrence [42]. In a systematic review of the treatment effectiveness for breast implant infection, CC was the most common complication following implant infection, as reported in several studies [43]. Hu and coworkers showed that bacterial count could be positively correlated with the degree of CC (*P* = 0.04) [44]. Miller and colleagues demonstrated that distant infection-induced hematogenous transmission of *Staphylococcus aureus* to the capsule in a rat model increased the peri-implant capsular thickness, number of myofibroblasts, and collagen density [45]. The microbial community associated with CC is diverse and highly variable [2]. Among them, *Staphylococcus epidermidis* (SES) is the most common bacterial species detected on the contracture capsule [46]. Notably, it is challenging for the host to eliminate SES by immune response as it forms the bacterial biofilm, leading to chronic inflammation persistently stimulating the capsule [47]. Xuan and coworkers activated the IL-6/STAT3 signaling pathway by continuous injection of lipoteichoic acid (LTA), a component of the cell wall of gram-positive bacteria. It induced prolonged inflammation, thus triggering capsular thickening, collagen deposition, and myofibroblast activation, ultimately resulting in CC. The administration of the IL-6 receptor antagonist, for instance, tocilizumab, could substantially relieve the CC [48].

#### Radiotherapy Affects CC

Typically, postmastectomy radiotherapy (PMRT) reduces local recurrence and improves survival in node-positive breast cancer. However, it increases the risk of CC occurrence in implant reconstruction patients. In a meta-analysis of patients with 1,234 reconstructed breasts, PMRT significantly raised the risk of wound infection (RR = 2.49) and CC (RR = 5.17). Radiotherapy often contributes to fibrosis by recruiting inflammatory cells and overexpressing Thy1 (CD90), promoting fibroblast activity and collagen deposition. In addition, CD26^+^ fibroblasts are more prevalent in the irradiated tissues, enhancing fibrotic potential. Inhibiting CD26 with diprotin A reduced scars in the animal models. Moreover, irradiation induces B cell infiltration, especially in irradiated capsular tissues, activating the Wnt signaling pathway and leading to increased synthesis of prostaglandin E2 and fibrosis. PMRT alters implant biomaterial properties at the nanoscale, producing debris that may promote CC. The irradiated tissues are more prone to ischemia and bacterial infection, contributing to CC. Further investigations are required to demonstrate the exact role of irradiation [49–58].

### Strategies to Prevent and Reduce CC

#### Selection of Implants and Surface Modification Techniques

Since the early 1960s, silicone has been widely used for soft tissue reconstruction. Nevertheless, high hydrophobicity and poor histocompatibility of silicone often lead to fibrous capsule formation, CC condition, and implant deformation sequentially. Various chemical and physical surface modification techniques have been developed to improve biocompatibility and reduce CC risk, influencing protein adsorption and foreign body reactions (FBRs) on implant surfaces. For instance, polyurethane-coated implants could reduce CC rates, which, however, resulted in chronic inflammation and breast implant (macrotextured)-associated anaplastic large cell lymphoma (BIA-ALCL). The textured implants are categorized by surface roughness (Ra), with smoother or nanotextured surfaces (< 5 μm) found to reduce inflammation and FBR.

Various surface modification techniques include plasma induction, UV/ozone treatment, and chemical coatings with hydrophilic polymers or antibiotics to prevent bacterial adhesion and protein adsorption. The electrospinning method has been used to create microstructured polydimethylsiloxane (PDMS) surfaces, promoting biocompatibility and reducing fibroblast differentiation. The multilayer coatings of poly-_L_-lysine and hyaluronic acid further inhibited CC. Moreover, modifying PDMS with itaconic acid (IA) and human adipose-derived stem cells (hASCs) could show promise in enhancing anti-fibrotic effects in an anti-inflammatory environment. Micropatterning (micropillars and micropits) has shown potential in preventing fibrotic capsule formation by regulating fibroblast differentiation. Several natural polymers (collagen) were combined with synthetic polymers to improve mechanical strength while maintaining biocompatibility and reducing fibrosis. PLGA coated with TGF-β inhibitors (tranilast) could provide sustained local drug release and effectively reduce fibrous capsule formation. The plasma treatments could transform the silicone surface from hydrophobic to hydrophilic, enhancing antimicrobial properties and reducing CC risk.

In another instance, carbon ion implantation combined with asiaticoside inhibited fibroblast activity and capsular formation. Polydopamine grafting with collagen-enhanced mesenchymal stem cell adhesion. Met-Z2-Y12 coatings and MPC-functionalized PDMS also reduced capsular thickness and FBR, while polycarboxybetaine prevented macrophage activation. UV treatments improved silicone surface wettability and hydrophilicity, offering the potential for long-term biocompatibility. These modifications provided promising strategies for enhancing implant safety and reducing the incidence of CC, requiring further research to optimize these methods for clinical use [10, 59–80].

#### Intraoperative Preventive Measures

During surgery, it is mandatory to follow aseptic procedures, treating the prosthesis in a noncontact manner to minimize contact between the implant and bacteria and thus prevent CC. In a case, Keller Funnel was effective for noncontact breast augmentation and reconstruction [81]. Hematoma, an important source of bacterial infection, worsens the postoperative inflammatory response, stimulating fibrous tissue proliferation and resulting in CC. Therefore, drainage tubes should be placed after surgery to prevent postoperative hematoma and reduce CC [32, 82]. Moreover, 32 SD rats were divided into 4 groups and injected with different doses of methylene blue, assessing its effect on periprosthetic CC. The results revealed that methylene blue injected around silicone implants promoted CC, in which the degree of CC was dose-independent [83].

#### Rise of Hybrid Breast Reconstruction (HBR)

HBR, a combination of IBBR and fat transplantation, has been proposed to improve implant coverage, treat local tissue defects, and redesign the shape of the breast [84]. The cell-assisted lipotransfer (CAL) technique is applied to mix adipose-derived stem cells (ADSCs) with fat grafts. CAL has been verified to improve fat graft survival, reduce fat necrosis, and raise skin coverage quality [85]. ADSCs possess anti-inflammatory and immunomodulatory effects, regenerative potential, and antibacterial properties, making them attractive in reconstructive surgery. Nevertheless, ADSCs may potentially promote tumor growth or metastasis, which remains to be explored [70, 84, 86]. In a case, PDMS and IA-PDMS samples cultured with human ADSCs showed that stem cells enhanced the anti-fibrosis ability of silicone implants and reduced CC [70]. Together, HBR could provide new ideas and strategies for preventing CC.

### Prevention and Mitigation Strategies

#### Selection of Incision and Surgical Procedures

Notably, CC increases with incisions closer to the nipple-areolar complex. Bresnick SD recommends periareolar incisions for limited breast tissue, vertical for tissue reduction, and inframammary fold for independent implants. Nevertheless, the axillary re-augmentation has shown positive outcomes. Patients receiving radiation to tissue expanders encounter more complications than those with permanent implants. However, immediate expander/implant reconstruction remains a viable option for the PMRT approach. Techniques like capsulectomy or capsulotomy prevent CC by creating more space for prostheses. A punctiform-incision approach minimizes tissue damage and improves outcomes for subpectoral implant CC. Transumbilical silicone breast augmentation (TUSBA) is a new technique with low complication rates. In a study, 40 women found no CC due to the incision’s distance from the nipple-areolar complex, reducing infection risk. However, TUSBA requires experience and time to improve the expertise [87–93]. As mentioned earlier, aseptic procedures should be strictly followed during surgery by treating the prosthesis in a noncontact manner to minimize contact between the implant and bacteria, thus preventing CC [32, 81–83].

#### Drug Usage

Several advancements in CC pathogenesis have led to the development of various drug-based prevention and treatment methods (Table 1). In a case, triamcinolone acetonide, a steroid, injected into the periprosthetic space under ultrasound guidance significantly reduced CC. The polyurethane mesh-loaded sustained-release implants and drug-delivery chips demonstrated anti-fibrosis effects in animal models. Several drugs (diclofenac and tranilast) exhibited promising potential in controlled delivery systems. PLGA microspheres effectively reduced fibrotic tissue formation through the controlled release of encapsulated Kynurenic acid. Several medical measures have been explored to prevent CC during surgery. The coagulase-negative *Staphylococci* and *Propionibacterium* have been identified as the main microorganisms associated with CC. Accordingly, preoperative antibiotics (cephalosporins and vancomycin) and doxycycline-coated silicone implants reduced biofilm formation and surgical site infections. In addition, irrigation with triple antibiotic (gentamicin/cefazolin/bacitracin) and hypochlorous acid (HOCl) showed the potential to prevent early infections and lower the incidence of CC [85, 94–98]^.^

**Table 1:** A summary of representative drugs reported for prevention and treatment of CC and their possible mechanisms

Drug	Mechanism of action	Therapeutic effect	References
*I. Anti-inflammatory/immunomodulatory drugs*			
Glucocorticoid			
Dexamethasone	Inhibits inflammation	NA [99]	[99, 100]
Triamcinolone		The capsular thickness decreased by 55.2% in the experimental group and increased by 61.8% in the control group	
Antihistamine drugs			
Roxatidine (histamine receptor-2 inhibitor)	Inhibits activation of NF κB and p38/MaPK signaling pathways in macrophages	NA	[101]
Nonsteroidal anti-inflammatory drugs (NSAIDs)			
Diclofenac (COX-2 inhibitor)	Inhibits cyclooxygenase (COX)	NA	[102]
Leukotriene receptor antagonists (LTRAs)			
Zafellukast	Inhibits cysteine leukotrienes and other potent inflammatory mediators	NA	[74, 103]
Montelukast			
*II. Anti-fibrotic drugs*			
TGF-β inhibitor			
Tranilast	Inhibits TGF-β-induced extracellular matrix synthesis	The capsular thickness in the experimental group decreased approximately 1.2-fold and 2.6-fold at 12 weeks. (Rat model)	[10]
CD26/ DPP4 inhibitors			
Diprotin A	Reduces scarring	NA	[53]
Pirfenidone	Inhibits fibroblast biological activity and inflammatory response	In the rabbit ear pocket model, the thickness of implants decreased significantly, like the intact dermis thickness	[104]
Halofuginone	Inhibits the NF-κB signaling pathway, as well as reduces inflammation and collagen deposition	NA	[105]
Asiaticoside	Inhibits the release of growth factors, promotes scar apoptosis, reduces immune cells, and relaxes collagen fibers	The levels of α-SMA and Col-1A1 in the experimental group decreased significantly	[75]
Kynurenic acid (KynA)	Inhibits collagen type I and fibronectin and promotes MMP expression	NA	[106]
Angiotensin-converting enzyme inhibitors (ACEIs)			
Ramipril	Reduces synthesis of TGF-β1	The capsular thickness, fibrosis rate, and TGF-b1 level in the experimental group were significantly lower than those in the control group	[107]
Omega-3 fatty acids	Destroy collagen deposits	Mean capsular thickness in the omega-3 group (rats) (205.09 μm) < control group (361.63 μm)	[108]
Botulin toxin type-A (BTX-A)	Reverses the effect of TGF-β1 and promotes fibroblast apoptosis	BTX-A could alleviate HS and CC by inhibiting the phenotypic transformation	[109]
*III. Antibiotics*			
Salinomycin	Bactericidal, bacteriostatic, and anticoccidial effects, and reduce constitutive Thy1 expression	NA	[3]
Itaconic acid	Inhibits bacteria and up-regulates IRG1	NA	[110]
Vancomycin/cefuroxime/rifampicin/minocycline	Bactericidal, bacteriostatic, and anti-inflammation effects	NA	[111]
*IV. Chemotherapy drugs*			
Paclitaxel	Inhibits HTFs, cell cycle, TGF-β1, and collagen matrix contraction	NA	[112]
*V. Endocrine drugs*			
Estrogen receptor antagonist Tamoxifen	Reduces proliferation and contraction of myofibroblasts and inhibits TGF-β1 production	Among patients undergoing endocrine therapy, tamoxifen was least associated with severe contracture (27.8%) and most significantly negatively related to the severity of contracture (*P* < 0.0001). In the mouse model, the capsular thickness in the treatment group decreased by 59% compared with the control group	[113, 114]

## Future Directions

In summary, several studies are required to focus on long-term, large-scale clinical trials, evaluating the effectiveness of novel implant materials, surgical techniques, and postsurgical interventions in reducing CC. In addition, understanding the molecular mechanisms underlying macrophage polarization and fibroblast activation may lead to the development of targeted therapies for preventing CC. Investigating the role of stem cells and biomaterials in tissue integration and fibrosis suppression also holds promise for advancing implant-based reconstruction techniques.

## Data Availability

The review data are available from the corresponding author upon request.
